# Vaccine efficacy in CKD patients not on dialysis: a systematic review and meta-analysis

**DOI:** 10.1093/ckj/sfag056

**Published:** 2026-02-19

**Authors:** Tanat Lertussavavivat, Suramath Isaranuwatchai, Somchai Eiam‐Ong, Kearkiat Praditpornsilpa, Paweena Susantitaphong

**Affiliations:** Department of Pharmacology, Faculty of Medicine, Chulalongkorn University, Bangkok, Thailand; Faculty of Medicine, King Mongkut’s Institute of Technology, Bangkok, Thailand; Division of Nephrology, Department of Medicine, Faculty of Medicine, King Chulalongkorn Memorial Hospital, Chulalongkorn University, Bangkok, Thailand; Division of Nephrology, Department of Medicine, Faculty of Medicine, King Chulalongkorn Memorial Hospital, Chulalongkorn University, Bangkok, Thailand; Division of Nephrology, Department of Medicine, Faculty of Medicine, King Chulalongkorn Memorial Hospital, Chulalongkorn University, Bangkok, Thailand; Center of Excellence for Metabolic Bone Disease in CKD Patients, Faculty of Medicine, King Chulalongkorn Memorial Hospital, Chulalongkorn University, Bangkok, Thailand

**Keywords:** chronic kidney disease, efficacy, seroconversion, seroprotection, vaccine

## Abstract

**Background:**

Patients with chronic kidney disease (CKD) have increased infection risk, contributing to higher rates of hospitalization and mortality. While vaccines can prevent or reduce the severity, data primarily focus on dialysis-dependent patients. This meta-analysis aimed to evaluate vaccine efficacy in non-dialysis CKD patients (CKD-ND).

**Methods:**

We systematically searched the MEDLINE, Embase, Scopus and OVID databases through April 2025 for randomized controlled and observational studies reporting the vaccine efficacy in stage 1–5 CKD-ND patients. Efficacy was assessed using laboratory markers (seroconversion, antibody titres) and clinical outcomes. The quality of the studies was assessed by ROBINS-I V2 and RoB2. A random effects model was used to estimate pooled effect size with 95% confidence interval.

**Results:**

Twenty-eight studies involving >500 000 participants were included covering hepatitis B virus (HBV), COVID-19, influenza, herpes zoster, pneumococcus and human papillomavirus (HPV) vaccines. Most studies were graded fair quality. Ten studies on HBV vaccination revealed a pooled seroconversion rate of 80% that decreased to 60% at 12 months post-vaccination. For COVID-19, the pooled anti-spike immunoglobulin G titre was 228.39 BAU/ml, with a reduced risk of COVID-19 infection following a boosting dose. Two doses of the influenza vaccine yielded higher seroconversion rates than a single dose, but the antibody levels declined over time, indicating waning immunity. The zoster vaccine showed a pooled adjusted hazard ratio of 0.74 for herpes zoster incidence compared with those who were unvaccinated. Pneumococcal vaccine elicited a modest transient response and was associated with reduced *Streptococcus pneumoniae* hospitalization and community-acquired pneumonia risk. The HPV vaccine demonstrated 100% seroconversions, although with lower HPV neutralizing antibody levels.

**Conclusion:**

Vaccination in CKD-ND patients is associated with a high rate of seroconversion and seroprotection across multiple vaccines. However, these surrogate markers may not fully reflect clinical effectiveness. Further studies are needed to evaluate the impact of vaccination on demonstrated clinical outcomes, particularly infection-related morbidities and mortalities.

## GRAPHICAL ABSTRACT

**GRAPHICAL ABSTRACT:**
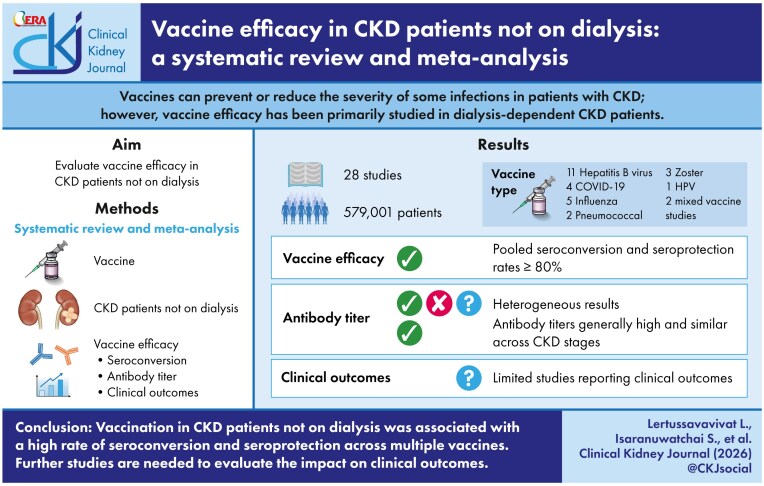

KEY LEARNING POINTS **What was known:**

- Chronic kidney disease (CKD) patients have impaired immunity and high infection risk, but most vaccine studies have focused on dialysis or transplant groups.
- Evidence in non-dialysis CKD (CKD-ND) is limited and fragmented.

**This study adds:**

- This is the first comprehensive meta-analysis (>500 000 patients) of vaccine efficacy in CKD-ND.
- Vaccines showed acceptable seroconversion/seroprotection, although immunity waned over time.
- Some vaccines (COVID-19, hepatitis B, pneumococcal) reduced infection risks.

**Potential impact:**

- Supports early, comprehensive vaccination in CKD-ND to lower infection burden.
- Highlights the need for boosters, higher doses or adjuvanted vaccines.
- Underscores gaps requiring large-scale trials to confirm clinical benefits.

## INTRODUCTION

Chronic kidney disease (CKD) is defined by abnormalities in kidney structure or function for >3 months [1]. It affects millions of people globally and contributes to all-cause mortality [2]. CKD patients are at increased risk of infection and infection-related mortality due to impaired immune function and immune senescence [3, 4]. Immunizations can prevent or reduce the severity of infections [5]. Accordingly, the Kidney Disease: Improving Global Outcomes (KDIGO) 2024 guidelines for the evaluation and management of CKD recommended incorporation of vaccination into the core CKD care models [1]. The current Advisory Committee on Immunization Practices (ACIP) also recommends that CKD patients receive specific vaccines, including influenza, tetanus–diphtheria–pertussis (DPT), varicella, human papillomavirus (HPV), herpes zoster virus (HZV), measles–mumps–rubella (MMR), pneumococcus and hepatitis B virus (HBV) [6].

Despite these recommendations, vaccine response in CKD patients is often suboptimal, particularly in those receiving kidney replacement therapy (KRT) [5, 7, 8]. In CKD, reduced vaccine efficacy is largely attributed to impaired humoral and cellular immunity in the setting of uraemia, oxidative stress, increased intestinal permeability and metabolic abnormalities such as erythropoietin and vitamin D deficiency, all of which contribute to immune dysfunction [5, 9, 10]. These pathophysiological changes, known as ‘inflammaging’, are characterized by premature immune system aging accompanied by chronic low-grade inflammation, leading to immune cell exhaustion and functional impairment [7]. Uraemic toxins further compromise immune processes, resulting in diminished vaccine-induced immunity [11]. In kidney transplantation (KT) recipients, immunosuppressive therapy further compromised vaccine efficacy [5, 12]. Despite the low vaccine response being well documented across the CKD patient spectrum, standardized vaccination in CKD patients is lacking [5]. Existing research has focused on vaccine efficacy and safety in CKD dialysis and KT populations [12–22], while data on non-dialysis CKD (CKD-ND) patients remain limited. Therefore, we conducted this meta-analysis to evaluate and update the current evidence on various vaccine efficacies in the CKD-ND population.

## MATERIALS AND METHODS

### Data sources and searches

This meta-analysis followed the Preferred Reporting Items for Systematic Reviews and Meta-Analyses (PRISMA) guidelines (Supplement 1). The MEDLINE, Embase, Scopus and OVID databases were searched through April 2025 to identify eligible studies. For the search, the following Medical Subject Headings (MeSH) search terms were used: ((Vaccine) AND (Efficacy)) AND ((chronic kidney disease) OR (dialysis) OR (end stage renal disease [MeSH Terms]) OR (end stage renal failure [MeSH Terms]) OR (end stage kidney disease [MeSH Terms]) OR (ESRD [Title/Abstract]) OR (end stage renal disease [Title/Abstract]) OR (end stage renal failure [Title/Abstract]) OR (end stage kidney disease [Title/Abstract])). The terms dialysis and ESKD were included in the search term for an extensive search result. Studies without a target population of CKD-ND will be excluded later. Reference lists and all prior systematic reviews and meta-analyses were searched manually to identify additional eligible studies. Meta-analysis was conducted using the PICO (patient/problem, intervention, comparison and outcome) framework to systematically formulate the research question, define inclusion/exclusion criteria and guide the entire study selection and synthesis process. The protocol was registered in PROSPERO (International Prospective Register of Systematic Reviews; ID 1057233).

### Eligibility criteria

We included studies that evaluated vaccine response specifically in CKD-ND patients. Eligible studies were required to report vaccine efficacy through at least one of the following outcomes: serologic response measured by antibody titres, rate of seroconversion or clinical effectiveness in preventing infection-related outcomes, such as incidence of infection, hospitalization or mortality associated with the target pathogen.

Studies involving patients on haemodialysis, peritoneal dialysis or those who had undergone KT were excluded. Only studies published in English and conducted in adult populations (age ≥18 years) were considered. Eligible study designs included cohort studies, case–control studies and cross-sectional studies. Meta-analyses, reviews, case studies, case reports, case series and letters were excluded.

### Study selection

Two authors (T.L. and S.I.) independently screened the titles and abstracts of all electronic citations and full-text articles were retrieved for a comprehensive review and independently rescreened. Each study was independently assessed by two authors (T.L. and S.I.). Disagreements were resolved through adjudication by a third author (P.S.).

### Data extraction

Two authors (T.L. and S.I.) independently extracted data from each included study using a standardized form. The following information was collected: first author’s name, year of publication, country of study, study design, number of cases and controls, participant characteristics [including age, sex and estimated glomerular filtration rate (eGFR)], vaccine type and subtype, vaccine regimen and duration of follow-up.

Vaccine efficacy data were also extracted for the pooled analysis. Vaccine efficacy was assessed through both laboratory and clinical outcomes. Laboratory outcomes included an increase in antibody titre following vaccination; seroprotection, defined as achieving the study-specific antibody threshold for protection against the infection; and seroconversion, defined as a change from negative to positive in protective antibody titres. Clinical outcomes encompassed reductions in infection rates, hospitalizations or mortality related to the target infection of the vaccine.

### Quality assessment

Two authors (T.L. and S.I.) independently assessed study quality with disagreements resolved by discussion and consensus was reached by a third author (P.S.). The quality of the included non-randomized studies was evaluated using version 2 of Risk Of Bias In Non-randomized Studies of Interventions (ROBINS-I V2) tool [23]. Randomized studies were evaluated by a revised tool to assess risk of bias in randomized trials (RoB 2) [24]. The overall risk was classified as poor, fair or good quality for each study. The Grading of Recommendations Assessment, Development, and Evaluation (GRADE) approach was used to evaluate the overall certainty of evidence, which was categorized as high, moderate, low or very low [25].

### Statistical analysis

Regarding available studies’ outcomes for meta-analysis, random effects models were used to generate a pooled effect size, which included serology titres and the odds ratio of seroconversion at a given threshold. Unadjusted and adjusted hazard ratios (HRs) of the interested clinical outcomes were also explored. All pooled estimates are provided with 95% confidence intervals (CIs). Heterogeneity was assessed using the *I*^2^ index and the *Q* test *P*-value, with a significant *I*^2^ index of ≥75%, indicating a medium–high heterogeneity. Publication bias was formally assessed using the Egger test [26].

The meta-analysis and meta-regression analyses were performed using Comprehensive Meta-Analysis version 2.0 (Biostat, Englewood, NJ, USA; https://www.meta-analysis.com) and the ‘metafor’ package in R version 2.14.0 (R Foundation for Statistical Computing, Vienna, Austria), respectively.

## RESULTS

**Figure 1:**
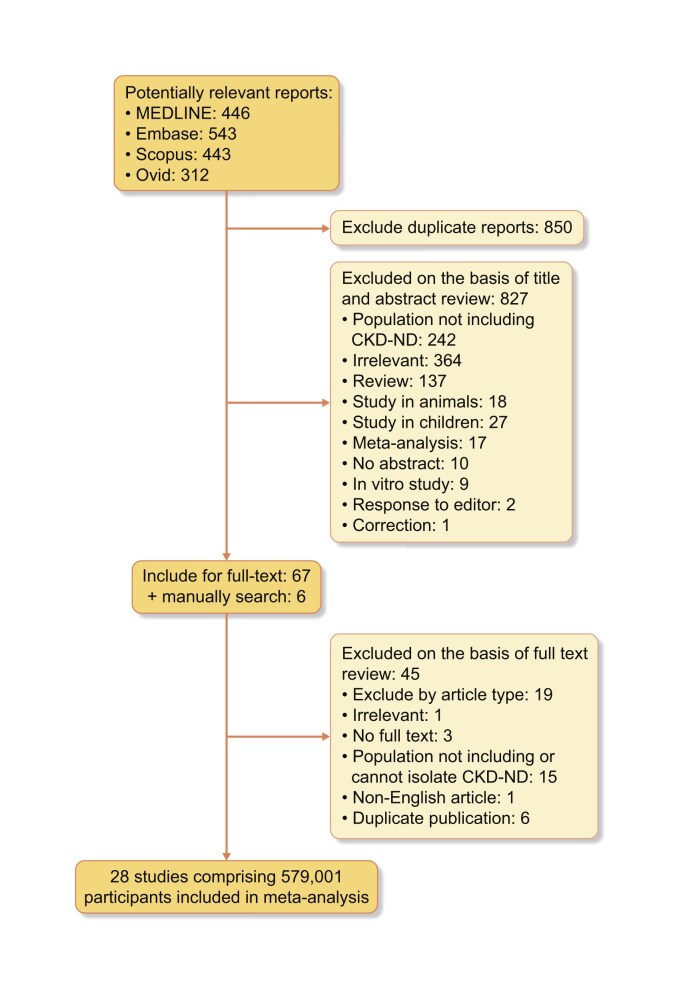
Flow chart of study selection.

A total of 1744 potentially relevant citations (446 from MEDLINE, 543 from Embase, 443 from Scopus and 312 from OVID) were identified and screened. A total of 67 articles were retrieved for detailed evaluation, of which 28 studies from >15 countries worldwide with 579 001 participants fulfilled eligibility criteria and were included in the meta-analysis (Fig. 1 and Table 1 ). Studies included vaccine efficacy in CKD-ND in HBV, coronavirus disease 2019 (COVID-19), influenza, HZV, pneumococcal and HPV. Most of the included studies used serological outcomes as the primary outcome; only nine studies used clinical outcomes as the primary outcome. A summary of the main findings, including heterogeneity assessment and the certainty of evidence evaluated by the GRADE approach for each vaccine outcome, is presented in Table 3. The risk of bias evaluation of all studies in this systematic review and meta-analysis by ROBIN-I V2 and RoB 2 is shown in Tables 3 and 4, respectively.

**Table 1:** Characteristics of the studies included in our systematic review and meta-analysis.

Author	Vaccine	Design	Country	Only CKD-ND, *n*	Age (years), median	Female (%)	eGFR (ml/min/1.73 m^2^), median	Healthy control	Duration of follow-up	Outcome
Hernán-García *et al*. [27]	HBV	RCo	Spain	173	ND	18.5	ND	No	36 months	Seroprotection
Fernández Sánchez-Escalonilla *et al*. [31]	HBV	PCo	Spain	195	ND	32.3	ND	No	7 months	Seroprotection
Kittrakulrat *et al*. [34]	HBV	RCT	Thailand	133	58.3	46.6	ND	No	52 weeks	Seroconversion
Fabrizi *et al*. [30]	HBV	PCo	Italy	107	66.3	27.5	20	No	12 months	Seroprotection
García-Agudo *et al*. [32]	HBV	PCo	Spain	155	65	33.6	28.7	No	6 months	Seroprotection
Hashemi *et al*. [33]	HBV	PCo	Iran	167	57.4	42.5	26.7	No	8 months	Seroconversion
Siddiqui *et al*. [35]	HBV	RCT	India	130	42.2	34.6	ND	Yes	6 months	Seroprotection
McNulty *et al*. [28]	HBV	RCT	UK	121	65	38	16	No	6 months	Seroconversion
Daroza *et al*. [37]	HBV	PCo	Canada	165	59.8	35.4	20	No	12 months	Seroconversion
Singh *et al*. [36]	HBV	RCT	India	63	45.7	38.1	18.3	No	6 months	Seroprotection
Seaworth *et al*. [29]	HBV	RCT	USA	61	45	26.2	ND	No	12 months	Seroconversion
Atiquzzaman *et al*. [40]	COVID-19	RCo	Canada	18 850	74	47	ND	No	>1 year	Infection rate
Bielopolski *et al*. [41]	COVID-19	RCo	Israel	67 861	76	52	44.76	Yes	2 months	Incidence rate
Trakarnvanich *et al*. [39]	COVID-19	PCo	Thailand	212	54.8	50.9	32	Yes	24 weeks	Antibody titre
Buchwinkler *et al*. [38]	COVID-19	PCo	Austria	582	63.1	41	29.9	No	120 days	Antibody titre
Heryaman *et al*. [43]	Influenza	PCo	Indonesia	67	53.2	73.1	61	Yes	1 month	Antibody titre
Uemura *et al*. [47]	Influenza	RCo	Japan	2017	ND	58.5	ND	No	4.9 years	Incidence rate
Chang *et al*. [42]	Influenza	PCo	Taiwan	198	64.1	40.9	ND	No	20 weeks	Seroprotection
Ishigami *et al*. [44]	Influenza	RCo	USA	31 959	75.5	58	ND	No	9 months	Incidence rate
Nikoskelainen *et al*. [46]	Influenza	PCo	Finland	15	ND	48	ND	Yes	8 weeks	Antibody titre
Wall *et al*. [48]	Influenza and pneumococcal	PCo	UK	61	75	30	21	Yes	6 months	Antibody titre
McDonald *et al*. [45]	Influenza and pneumococcal	RCo	UK	67 435	72	49	ND	No	>5 years	Incidence rate
Le *et al*. [53]	Pneumococcal	RCo	Canada	1455	69	48	43.1	No	5 years	Incidence rate
Fuchshuber *et al*. [52]	Pneumococcal	PCo	Germany	58	12.9	ND	ND	No	1 year	Antibody level
Oostvogels *et al*. [51]	HZV	RCo	Multiple	27 916	68.5	57.9	ND	Yes	90 days	Infection rate
Izurieta *et al*. [50]	HZV	PCo	USA	175 023	77	67	ND	No	>4 years	Infection rate
Langan *et al*. [49]	HZV	RCo	USA	183 762	ND	67.7	ND	No	n/a	Incidence rate
Praditpornsilpa *et al*. [54]	HPV	PCo	Thailand	3	21.5	53.3	14.3	No	6 months	Seropositivity

Mod: moderate; n/a: not applicable; ND: no data; PCo: prospective cohort; RCo: retrospective cohort.

**Table 2:** Summary of results and evidence quality: the GRADE framework.

Vaccine	Outcome	Timing	Participants (studies), *n*	Result (95% CI)	Heterogeneity, %	*P*-value	Certainty
HBV	Seroconversion rate	6 months	1796 (15)	80% (95% CI 70.4–87.1)	88.2%	<.001	⊕⊕⊕◯ Moderate, due to inconsistency
		12 months	377 (4)	60.6% (95% CI 28.5–85.6)	89.09	<.001	⊕⊕⊕◯ Moderate, due to inconsistency
COVID-19	Antibody titre	3 months	172 (2)	228.39 BAU/ml (95% CI 152.74–304.03)	53.02	.014	⊕◯◯◯ Very low, due to inconsistency
Influenza A H1N1	Seroconversion rate	1 months	214 (3)	92.758 (95% CI: 87.670–98.141)	0	.763	⊕⊕◯◯ Low
		2 months	215 (3)	92.758 (95% CI: 87.670–98.141)	0	.763	⊕⊕◯◯ Low
		5 months	216 (3)	87.124 (95% CI: 81.030–93.677)	0	.847	⊕⊕◯◯ Low
Influenza B	Seroconversion rate	1 months	217 (3)	80.028 (95% CI: 82.080–94.406)	0	.864	⊕⊕◯◯ Low
		2 months	218 (3)	88.959 (95% CI: 81.440–97.172)	25.80	.25	⊕⊕◯◯ Low
		5 months	219 (3)	86.500 (95% CI: 79.250–94.413)	44.70	.108	⊕⊕◯◯ Low
HZV	Adjusted HR	≈2.5 years	358 785 (2)	0.744 (95% CI: 0.374–1.478)	92.56	<.001	⊕◯◯◯ Very low, due to indirectness of evidence

**Table 3:** Risk of bias in non-randomized studies included in our systematic review and meta-analysis.

Authors	Vaccine	Risk of bias (using ROBIN-I V2)^a^							Overall judgement
		D1	D2	D3	D4	D5	D6	D7	
Hernán-García *et al*. [27]	HBV	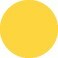	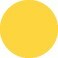	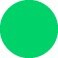	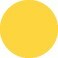	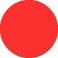	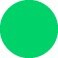	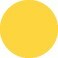	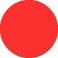
Fernández Sánchez-Escalonilla *et al*. [31]	HBV	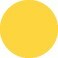	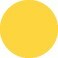	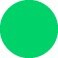	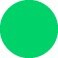	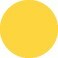	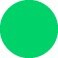	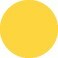	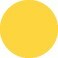
Fabrizi *et al*. [30]	HBV	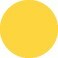	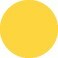	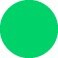	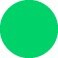	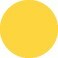	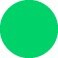	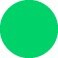	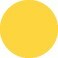
García-Agudo *et al*. [32]	HBV	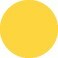	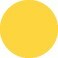	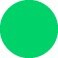	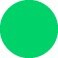	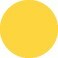	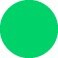	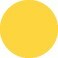	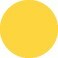
Hashemi *et al*. [33]	HBV	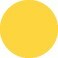	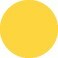	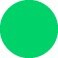	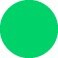	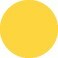	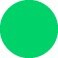	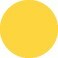	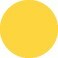
Daroza *et al*. [37]	HBV	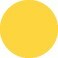	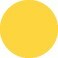	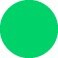	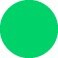	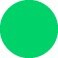	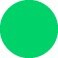	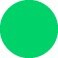	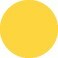
Atiquzzaman *et al*. [40]	COVID-19	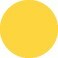	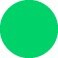	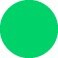	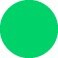	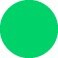	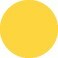	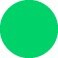	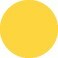
Bielopolski *et al*. [41]	COVID-19	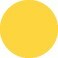	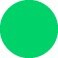	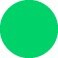	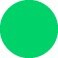	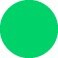	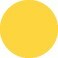	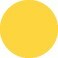	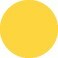
Trakarnvanich *et al*. [39]	COVID-19	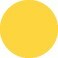	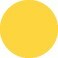	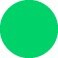	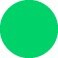	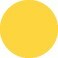	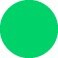	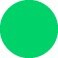	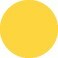
Buchwinkler *et al*. [38]	COVID-19	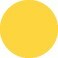	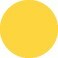	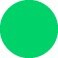	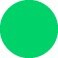	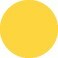	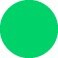	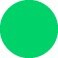	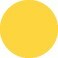
Heryaman *et al*. [43]	Influenza	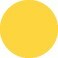	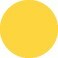	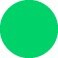	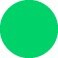	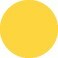	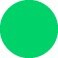	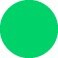	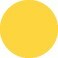
Uemura *et al*. [47]	Influenza	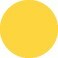	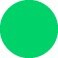	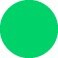	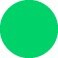	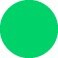	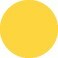	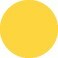	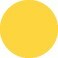
Chang *et al*. [42]	Influenza	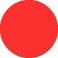	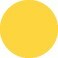	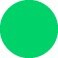	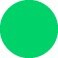	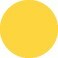	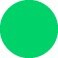	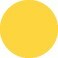	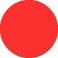
Ishigami *et al*. [44]	Influenza	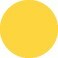	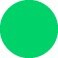	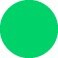	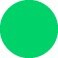	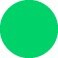	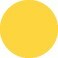	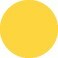	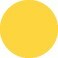
Nikoskelainen *et al*. [46]	Influenza	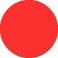	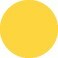	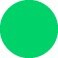	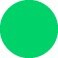	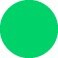	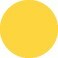	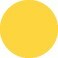	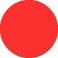
Wall *et al*. [48]	Influenza and pneumococcal	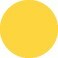	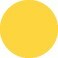	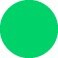	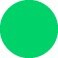	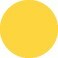	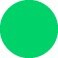	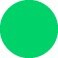	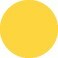
McDonald *et al*. [45]	influenza and pneumococcal	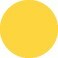	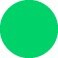	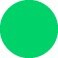	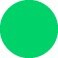	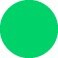	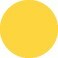	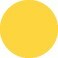	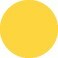
Le *et al*. [53]	Pneumococcal	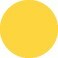	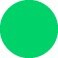	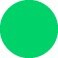	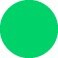	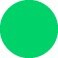	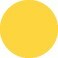	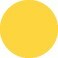	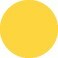
Fuchshuber *et al*. [52]	Pneumococcal	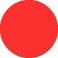	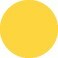	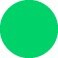	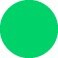	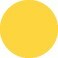	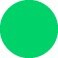	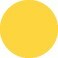	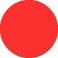
Oostvogels *et al*. [51]	HZV	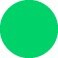	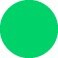	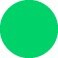	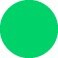	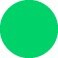	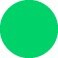	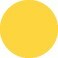	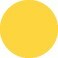
Izurieta *et al*. [50]	HZV	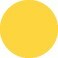	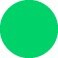	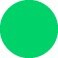	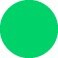	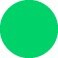	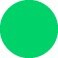	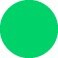	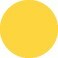
Langan *et al*. [49]	HZV	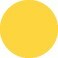	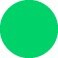	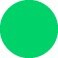	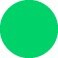	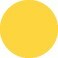	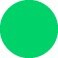	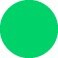	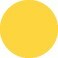
Praditpornsilpa *et al*. [54]	HPV	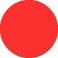	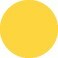	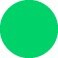	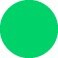	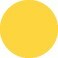	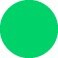	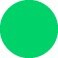	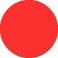

a Risk of bias was evaluated by the tool ROBIN-I V2, which evaluate the study in seven domains, including D1: bias due to confounding; D2: bias due to selection of participants; D3: bias in classification of interventions; D4: bias due to deviations from intended interventions; D5: bias due to missing data; D6: bias in measurement of outcomes; and D7: bias in selection of the reported result. The traffic light indicates the following: red: poor quality; yellow: fair quality; green: good quality.

**Table 4:** Risk of bias in randomized studies included in our systematic review and meta-analysis.

Authors	Vaccine	Risk of bias (using RoB 2)^a^					Overall judgement
		D1	D2	D3	D4	D5	
Kittrakulrat *et al*. [34]	HBV	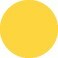	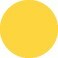	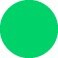	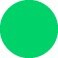	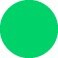	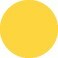
Siddiqui *et al*. [35]	HBV	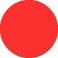	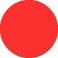	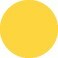	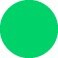	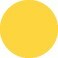	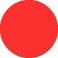
McNulty *et al*. [28]	HBV	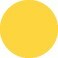	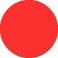	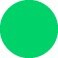	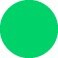	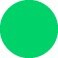	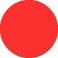
Singh *et al*. [36]	HBV	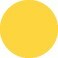	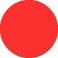	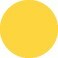	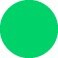	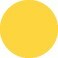	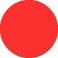
Seaworth *et al*. [29]	HBV	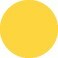	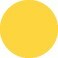	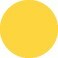	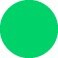	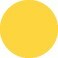	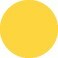

a Risk of bias was evaluated by the RoB 2 tool, which evaluates the study in five domains (D), including D1: randomization process; D2: deviations from intended interventions; D3: missing outcome data; D4: measurement of the outcome; and D5: selection of the reported result. The traffic light indicates the following: red: poor quality; yellow: fair quality; green: good quality.

### HBV vaccine

**Figure 2:**
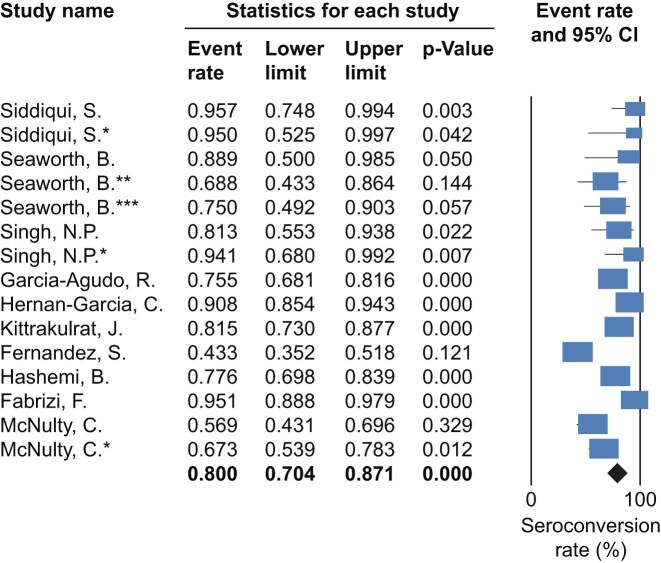
Forest plot of studies reporting HBV vaccine efficacy in CKD-ND.

There were 10 studies evaluating the percentage of seroconversion in CKD-ND patients after being vaccinated for 6 months [27–36]. In all studies, patients were regarded as responders if the hepatitis B surface antibody titre was >10 IU/l. The pooled percentage of seroconversion was 80% (95% CI 70.4–87.1), with an *I*^2^ of 88.25 (*P* < .001) (Fig. 2, Supplementary Table S1). At 12 months after vaccination, two studies reported fewer seroconverted patients [60.6% (95% CI 28.5–85.6)], with an *I*^2^ of 89.09 (*P* < .001) [29, 37]. These studies varied in vaccination regimen and type of vaccine (recombinant or plasma-derived, with or without adjuvant). A funnel plot was performed to assess publication bias (Supplementary Fig. S1).

In the subgroup analysis stratified by vaccine dosage, the high-dose group (40 μg, 10 studies) yielded a pooled event rate of 79.8% (95% CI 73.3–85.1). The low-dose group (20 μg, 5 studies) showed a similar pooled event rate of 80.9% (95% CI 53.4–94). The test for subgroup differences found no statistically significant difference in efficacy between the high-dose and low-dose regimens (*Q* = 0.01, *P* = .921). When restricted to studies using recombinant vaccines, the analysis included 10 studies and yielded a pooled seroconversion rate of 75.3% (95% CI 68.4–81.2). Heterogeneity remained moderate in this subgroup (*I*^2^ = 55.02%, *P* = .018).

In the subgroup analysis stratified by study design (Supplementary Tables S2–5), the cohort studies yielded a pooled event rate of 80.6% (95% CI 60.8–91.8). The randomized controlled trials (RCTs) showed a similar pooled event rate of 78.2% (95% CI 69.6–84.9). In a sensitivity analysis excluding poor-quality studies, the cohort group (four studies) yielded a pooled event rate of 76.9% (95% CI 54.2–90.3). The RCT group (four studies) showed a comparable pooled event rate of 79.5% (95% CI 72.1–85.3). The test for subgroup differences found no statistically significant difference between the high-quality cohort and RCT group (*Q* = 0.73, *P* = .787).

### COVID-19 vaccine

Two studies reported the efficacy of COVID-19 vaccination in CKD-ND patients. Buchwinkler *et al*. [38] reported an antispike immunoglobulin G (IgG) of 230.3 BAU/ml after two doses of messenger RNA (mRNA) vaccine in CKD-ND patients, while Trakarnvanich *et al*. [39] reported an antispike IgG of 150.13 BAU/ml after two doses of inactivated COVID vaccine. The pooled antispike IgG titre was 228.39 BAU/ml (95% CI 152.74–304.03 BAU/ml), with an *I*^2^ of 54.02 (*P* = .14) (Supplementary Table S6). A study by Atiquzzaman *et al*. [40] reported a decreasing risk of COVID-19 after a subsequent boosting dose, with an HR for COVID infection of 0.41, 0.29 and 0.22 after one, two and three doses, respectively.

In addition, a large observational study by Bielopolski *et al*. [41] reported that the clinical outcomes of two doses of the BNT162b2 mRNA vaccine in CKD patients varied significantly by CKD stage. Patients with mild to moderate CKD (stages 3A–3B; eGFR 30–59 ml/min/1.73 m^2^) showed no significant difference in COVID-19 outcomes compared with matched controls. However, those with advanced CKD (stages 4–5; eGFR <30 ml/min/1.73 m^2^) had markedly increased risks of severe COVID-19 [risk ratio (RR) 6.42 (95% CI 1.85–17.51)] and COVID-related death [RR 8.81 (95% CI 1.63–13.81)] compared with vaccinated matched controls without CKD.

### Influenza vaccine

Seven studies reported the efficacy of influenza vaccination in CKD-ND patients with different primary outcomes for effectiveness and methods to control for confounding [42–48]. In the studies that reported post-vaccination serology titre, the pooled seroconversion rate at 1 month post-vaccination was 92.8% (95% CI 87.7–98.1) for influenza A H1N1 and 80% (95% CI 82.1–94.4) for influenza B [42, 43, 48]. Patients who obtained two doses of the influenza vaccine showed higher seroconversion rates compared with those receiving a single dose. When stratified by CKD stage, patients with advanced CKD (stages 4–5) did not demonstrate inferior immune responses when compared with patients in earlier CKD stages (1–3) (Supplementary Tables S7 and S8). However, Ishigami *et al*. [44] and McDonald *et al*. [45] reported that in older CKD-ND (age >65 years) there were trivial differences in vaccination prevalence across eGFR strata. The antibody levels generally declined over time, with notable waning immunity observed at 20 weeks post-vaccination, consistent with reduced durability of seroprotection in CKD patients. However, the Egger’s test indicated potential publication bias (*P* < .05), supporting the concern that available data may be insufficient.

### HZV vaccine

By meta-analysis of two retrospective cohort studies, the pooled adjusted HR was 0.744 (95% CI 0.374–1.478) for HZV incidence prevention compared with unvaccinated patients [49, 50] (Supplementary Table S9). The heterogeneity was high (*I*^2^ = 92.6%). In the only study reported, HZV vaccine in terms of disease prevention showed an efficacy of 86.60% (95% CI 4.5–99.7) within 3 years of follow-up [51]. Of note, both studies evaluated a single-dose live attenuated HZV vaccine that has since been replaced in clinical practice by the recombinant HZV vaccine due to superior and more sustained immunogenicity.

### Pneumococcal vaccine

Four studies evaluated the immune response and clinical effectiveness of pneumococcal vaccination in patients with CKD-ND. Due to substantial heterogeneity in study designs, outcome measures and follow-up durations, a meta-analysis could not be performed (Supplementary Table S10). Overall, most studies suggest a modest and transient antibody response among CKD-ND patients. In a study by Fuchshuber *et al*. [52], the 23-valent pneumococcal vaccine (PPV23) was administered to 40 pre-dialysis patients. Four weeks after vaccination, 88% of the patients reached protective antibody titres, but they declined significantly within 6 months. Another study by Wall *et al*. [48] compared the efficacy of PPV23 between patients with CKD-ND and age- and sex-matched controls. Both groups exhibited modest responses to the vaccine, with only 12–18% of CKD patients showing adequate responses compared with 25% in controls.

In terms of clinical effectiveness, large-scale observational data provided additional insights. Le *et al*. [53], using administrative claims data, assessed vaccine effectiveness (VE) of PCV-13 and PPSV23 vaccines in individuals with and without reduced eGFRs. The adjusted population PCV13 VE was 39% (95% CI 13–8%) and the combination PCV13 and PPSV23 VE was 39% (95% CI 12–58). The PPSV23 VE was −3.7% (95% CI −57–32). Stratified by eGFR, adjusted PCV13 VE was consistent in eGFR ≥60 ml/min/1.73 m^2^ [VE 38% (95% CI 2.9–61)] and 30–59 ml/min/1.73 m^2^ [VE 61% (95% CI 24–80)] without significant interaction. PCV13 vaccination was associated with a reduced risk of *Streptococcus pneumoniae* hospitalization in individuals with a reduced eGFR (30–59 ml/min/1.73 m^2^).

McDonald *et al*. [45] reported a VE of 22% (95% CI 11–31) for PPSV23 against community-acquired pneumonia within the first year after vaccination. However, this protective effect declined substantially, becoming negligible after 5 years.

### HPV vaccine

There was only one study about the efficacy of the HPV vaccine in CKD-ND patients [54] and the CKD-ND subgroup comprised just three participants. Consequently, due to this extremely limited sample size, the data lack the statistical power required for meaningful clinical interpretation or meta-analysis.

## DISCUSSION

This study represents one of the first systematic reviews and meta-analyses focused specifically on vaccine efficacy in CKD-ND patients, with 28 studies and >500 000 participants worldwide. Overall, our findings suggest that vaccine efficacy in CKD-ND patients is generally acceptable, with pooled seroconversion and seroprotection rates >80%.

Ten studies addressed HBV vaccine efficacy, which is particularly important in CKD patients who may eventually require KRT. Achieving maintained HBV protection among CKD-D patients is a well-known challenge [55]. However, our study revealed that CKD-ND patients showed promising immune responses with seroconversion and seroprotection of ≈80% (anti-HBs >10 IU/l), although these immune responses decreased to 60% at 12 months post-vaccination. Currently, no standardized HBV vaccination regimen exists for CKD patients across the CKD spectrum. Both KDIGO and CDC Kidney Disease Work Group guidelines recommend initiating HBV vaccination when eGFR falls below 60 ml/min/1.73 m^2^ as immune responsiveness declines with worsening renal function [1].

For vaccines against respiratory viruses, including the COVID-19, influenza and pneumococcal vaccines, immunization was still associated with measurable antibody production. However, heterogeneity was high due to differences in vaccine type, brand, dosing and pre-existing immunity. Our results showed that the absolute antibody titres were generally high and comparable between early and advanced CKD stages, and antibody levels decreased over time, suggesting the potential need for higher antigen doses, adjuvanted formulations or more frequent boosters in this population [56].

Pneumococcal vaccines induced modest immune responses in CKD-ND patients, with antibody titres declining substantially within 6 months. These findings mirror the responses to pneumococcal vaccination in dialysis patients [57, 58]. Sequential administration of PCV13 followed by PPSV23 demonstrated higher vaccine effectiveness, particularly in patients with an eGFR of 30–59 ml/min/1.73 m^2^. Further research is warranted to assess the benefit of conjugate or sequential vaccination strategies in the CKD-ND population.

In our study, HZV vaccine efficacy was 87% during the first 3 years, while the HPV vaccine showed a 100% seroconversion rate in the very small pre-dialysis cohort. However, these findings should be interpreted cautiously due to limited sample sizes. Large population-based studies are required before recommendations can be made.

The lower vaccine efficacy in CKD-ND is likely multifactorial, including impaired antigen-presenting cell function due to uraemic toxins, T cell exhaustion and senescence, chronic low-grade inflammation or inflammaging, dysregulated B cell maturation and concomitant immunosuppressive medications [56, 59].

Moreover, vaccination rates among CKD patients remain unsatisfactory. Studies reveal that a significant portion of participants, ≈61.9% for influenza and 60.9% for pneumococcus, have never been vaccinated [60, 61]. The main barriers were limited knowledge about the vaccines and the lack of recommendations from healthcare providers [62]. To improve vaccination rates, policies should address factors that cause vaccine hesitancy and involve nephrologists in planning. Educational programs to improve vaccine understanding, along with simple, system-based tools such as nurse-led checklists or electronic health record reminders, can also help ensure vaccines are given on time [63].

Vaccination in the CKD-ND population is generally considered safe and well tolerated. Adverse events are typically mild and self-limiting, with reported incidence rates ranging from 11% to 50% [28, 29, 34]. These events most commonly manifest as local reactions, such as injection site pain, or transient systemic symptoms like headache and asthenia, as observed in studies of adjuvanted HBV and non-adjuvanted influenza vaccines.

It is very important to note that serology endpoints such as seropositivity and seroconversion do not always correlate with clinical protection [5, 64]. The primary goal of vaccination is to prevent infection or reduce the severity or mortality if infection occurs [65, 66]. However, measuring hard clinical outcomes in clinical trials is often challenging. Consequently, most studies rely on serology markers, which are easier to quantify. Most of the studies regarding vaccination in CKD-ND patients did not explore clinical outcomes, except several COVID-19 studies, which reported a reduced infection rate in vaccinated patients and comparable outcomes between patients with mild–moderate CKD and those without CKD. However, patients with advanced CKD had worse clinical outcomes [38, 39]. More multinational prospective studies in other vaccines are needed to determine whether vaccination in CKD-ND patients leads to improved clinical outcomes and to explore interventions to enhance immunogenicity in this population.

Although recent data remain incomplete regarding infection-related morbidities and mortalities, this study provides the first systematic review and meta-analysis of vaccine efficacy. We included studies with a large number of participants from multinational countries and conducted meta-analysis where methodology heterogeneity allowed.

Admittedly there were certain limitations in our study. The primary limitation of this study is that the majority of the included studies reported serologic endpoints as surrogate markers of immune response rather than clinical effectiveness, such as protection against symptomatic infection, hospitalization or mortality. While serological response is a standard measure of immunogenicity, it does not fully reflect clinical protection. Future studies incorporating both immunologic and clinical outcomes would therefore be valuable to validate and extend our findings.

Second, high heterogeneity was observed among the included studies due to differences in vaccine type, formulation and dosage schedules, which often varied across regions depending on local vaccination policies and product availability. Although we attempted to mitigate this limitation by performing subgroup and sensitivity analyses, residual confounding arising from these interstudy differences cannot be fully excluded. Furthermore, the body of evidence is predominantly based on data from HBV vaccine studies. In contrast, there remains a significant lack of data in the CKD-ND population for other clinically important vaccines, such as HPV and HZV. This skew in the available literature limits the generalizability of our conclusions to a broader spectrum of immunizations.

Third, we were unable to directly compare vaccine efficacy between patients with CKD-ND and those receiving dialysis, as only a limited number of studies provided head-to-head comparisons across these populations. Consequently, our findings should be interpreted as reflecting vaccine responses within the CKD-ND population rather than across the spectrum of CKD severity.

Fourth, a significant number of the included studies did not have healthy participants or did not have CKD-ND patients without vaccination as a control group. This lack of a control group substantially diminished data quality and interpretability in our systematic review and meta-analysis. We also further suggest that future studies about vaccination in CKD-ND patients should include control groups to compare the vaccine efficacy.

Lastly, evidence concerning less common vaccines in CKD is currently limited primarily to patients with advanced disease, such as those undergoing dialysis or KT, highlighting a significant lack of data for the CKD-ND population. For example, studies on the yellow fever vaccine (17DD-YF) in dialysis patients suggest it is safe and well-tolerated but is associated with poor immunogenicity, with only 38% of participants achieving an adequate serological response [67]. Similarly, hepatitis A vaccination in kidney transplant recipients has been found to be safe but exhibited reduced efficacy [68]. Further studies are needed to explore the safety and immunogenicity of these less common vaccines specifically in the CKD-ND population.

## CONCLUSION

Our systematic review and meta-analysis on vaccine efficacy in CKD-ND patients showed vaccination in is associated with acceptable immunogenicity across several commonly recommended vaccines. However, these findings are primarily based on serological response rates; the data on clinical effectiveness remain limited. Further large-scale prospective studies are needed to evaluate infection-related outcomes and establish an optimized vaccine strategy within high-risk population.

## Supplementary Material

sfag056_Supplemental_Files

## Data Availability

The datasets used and/or analysed during the current study are available from the corresponding author upon reasonable request.
