# Supplementary material for: Vaccine efficacy in CKD patients not on dialysis: a systematic review and meta-analysis
Source: Clin Kidney J. 2026 Feb 19;19(5):sfag056. doi: 10.1093/ckj/sfag056 (PMC13133630; doi:10.1093/ckj/sfag056)
Supplement: sfag056_Supplemental_Files [file sfag056_supplemental_files.zip › Supplementary Result Meta Vaccine CKD 08012026.docx]

**Supplementary Document for Efficacy of Various Vaccines in Non-dialysis Chronic Kidney Disease Patients: A Systematic Review and Meta-analysis**

Supplementary Table S1: Pooled Efficacy of Hepatitis B Vaccination in Non-Dialysis CKD Patients: Seroconversion Rates by Study and Timepoint

Supplementary Table S2: Pooled Efficacy of Hepatitis B Vaccination in Non-Dialysis CKD Patients: Subgrouped by Dosage (High dose 40 mcg and Low dose 20 mcg)

Supplementary Table S3: Pooled Efficacy of Hepatitis B Vaccination in Non-Dialysis CKD Patients: Only Recombinant Vaccine

Supplementary Table S4: Pooled Efficacy of Hepatitis B Vaccination in Non-Dialysis CKD Patients: Subgrouped by Study Type

Supplementary Table S5: Sensitivity Analysis of Pooled Efficacy of Hepatitis B Vaccination in Non-Dialysis CKD Patients: Only RCT (Exclude Poor Quality Studies)

Suppplementary Table S62: COVID-19 Vaccine Effectiveness in Non-Dialysis CKD Patients

Supplementary Table S7: Pooled Efficacy of Influenza A H1N1 Vaccination in Non-Dialysis CKD Patients: Seroconversion Rates with Subgroup by CKD Stage and Timepoint

Supplementary Table S8: Pooled Efficacy of Influenza B Vaccination in Non-Dialysis CKD Patients: Seroconversion Rates with Subgroup by CKD Stage and Timepoint

Supplementary Table S9: Pooled Efficacy of Herpes Zoster Vaccination in Non-Dialysis CKD Patients

Suppplementary Table S10: Pneumococcal Vaccine Seroprotection in Non-Dialysis CKD Patients

Supplementary Figure S1: Funnel Plot of Studies Reporting HBV Vaccine Efficacy in CKD-ND

**Supplementary Table S1: Pooled Efficacy of Hepatitis B Vaccination in Non-Dialysis CKD Patients: Seroconversion Rates by Study and Timepoint**

| **Author** | **N seroconvert** | **N  total** | **Seroconvert threshold** | **Timing (mo)** | **Vaccine** |
| --- | --- | --- | --- | --- | --- |
| Siddiqui S | 22 | 23 | ≥ 10 | 6 | 20 mcg of recombinant vaccine |
| Siddiqui S | 9 | 9 | ≥ 10 | 6 | 40 mcg of recombinant vaccine |
| Seaworth B | 8 | 9 | ≥ 10 | 7 | 20 mcg of recombinant vaccine |
| Seaworth B | 11 | 16 | ≥ 10 | 7 | 40 mcg of recombinant vaccine |
| Seaworth B | 12 | 16 | ≥ 10 | 7 | 40 mcg of plasma-derived vaccine |
| Singh NP | 13 | 16 | ≥ 10 | 7 | 40 mcg of recombinant vaccine |
| Singh NP | 16 | 17 | ≥ 10 | 7 | 40 mcg of recombinant vaccine + GCSF |
| Garcia-Agudo R | 117 | 155 | ≥ 10 | 7 | 40 mcg of recombinant vaccine |
| Hernan-Garcia C | 157 | 173 | ≥ 10 | 7 | 20 mcg of antigen with adjuvanted |
| Kittrakulrat J | 88 | 108 | ≥ 10 | 7 | 40 mcg of recombinant vaccine |
| Fernandez Sanchez-Escalonilla S | 58 | 134 | ≥ 10 | 7 | 20 mcg of antigen with adjuvanted |
| Hashemi B | 104 | 134 | ≥ 10 | 7 | 40 mcg of recombinant vaccine |
| Fabrizi F | 97 | 102 | ≥ 10 | 7 | 20 mcg of antigen with adjuvanted |
| McNulty C | 29 | 51 | ≥ 10 | 7 | 20 mcg of recombinant vaccine |
| McNulty C | 37 | 55 | ≥ 10 | 7 | 40 mcg of recombinant vaccine |
| **Efificacy at 6-7 months** | | | **I^2^** | **p-value** | **egger's test** |
| 15 studies arm | 80% (70.4-87.1) | <0.001 | 88.246 | <0.001 | 0.101 |
|  | | | | | |
| **Author** | **N seroconvert** | **N  total** | **Seroconvert threshold** | **Timing (mo)** | **Vaccine** |
| Seaworth B | 3 | 18 | ≥ 10 | 12 | 20 mcg of recombinant vaccine |
| Seaworth B | 9 | 17 | ≥ 10 | 12 | 40 mcg of recombinant vaccine |
| Seaworth B | 13 | 16 | ≥ 10 | 12 | 40 mcg of plasma-derived vaccine |
| DaRoza G | 136 | 165 | ≥ 10 | 12 | 40 mcg of recombinant vaccine or 40 mcg of plasma-derived vaccine |
| **Efificacy at 12 months** | | | **I^2^** | **p-value** | **egger's test** |
| 4 studies arm | 60.6% (28.5, 85.6) | 0.532 | 89.089 | <0.001 | 0.26 |

**Supplementary Table S2: Pooled Efficacy of Hepatitis B Vaccination in Non-Dialysis CKD Patients: Subgrouped by Dosage (High dose 40 mcg and Low dose 20 mcg)**

| **Author** | **N seroconvert** | **N** | **Seroconvert threshold** | **Timing (mo)** | **Vaccine** |
| --- | --- | --- | --- | --- | --- |
| Siddiqui S | 9 | 9 | ≥ 10 | 6 | 40 mcg of recombinant vaccine |
| Seaworth B | 11 | 16 | ≥ 10 | 7 | 40 mcg of recombinant vaccine |
| Seaworth B | 12 | 16 | ≥ 10 | 7 | 40 mcg of plasma-derived vaccine |
| Singh NP | 13 | 16 | ≥ 10 | 7 | 40 mcg of recombinant vaccine |
| Singh NP | 16 | 17 | ≥ 10 | 7 | 40 mcg of recombinant vaccine + GCSF |
| Garcia-Agudo R | 117 | 155 | ≥ 10 | 7 | 40 mcg of recombinant vaccine |
| Hernan-Garcia C | 157 | 173 | ≥ 10 | 7 | 20 mcg of antigen with adjuvanted |
| Kittrakulrat J | 88 | 108 | ≥ 10 | 7 | 40 mcg of recombinant vaccine |
| Hashemi B | 104 | 134 | ≥ 10 | 7 | 40 mcg of recombinant vaccine |
| McNulty C | 37 | 55 | ≥ 10 | 7 | 40 mcg of recombinant vaccine |
| **High dose (40 mcg)** | |  | **i2** | **p-value** |  |
| 10 studies arm | 79.8% (73.3-85.1) | <0.001 | 62.29 | 0.005 |  |
|  | | | | | |
| **Author** | **N seroconvert** | **N** | **Seroconvert threshold** | **Timing (mo)** | **Vaccine** |
| Siddiqui S | 22 | 23 | ≥ 10 | 6 | 20 mcg of recombinant vaccine |
| Seaworth B | 8 | 9 | ≥ 10 | 7 | 20 mcg of recombinant vaccine |
| Fernandez Sanchez-Escalonilla S | 58 | 134 | ≥ 10 | 7 | 20 mcg of antigen with adjuvanted |
| Fabrizi F | 97 | 102 | ≥ 10 | 7 | 20 mcg of antigen with adjuvanted |
| McNulty C | 29 | 51 | ≥ 10 | 7 | 20 mcg of recombinant vaccine |
| **Low dose (20 mcg)** | |  | **i2** | **p-value** |  |
| 5 studies arm | 80.9% (53.4-94) | 0.031 | 92.68 | 0 |  |
|  | | | | | |
| **Test for heterogeneity between groups** | | | **Q=0.010, df=1, p=0.921** | | |

**Supplementary Table S3: Pooled Efficacy of Hepatitis B Vaccination in Non-Dialysis CKD Patients: Only Recombinant Vaccine**

| **Author** | **N seroconvert** | **N** | **Seroconvert threshold** | **Timing (mo)** | **Vaccine** |
| --- | --- | --- | --- | --- | --- |
| Siddiqui S | 9 | 9 | ≥ 10 | 6 | 40 mcg of recombinant vaccine |
| Seaworth B | 11 | 16 | ≥ 10 | 7 | 40 mcg of recombinant vaccine |
| Singh NP | 13 | 16 | ≥ 10 | 7 | 40 mcg of recombinant vaccine |
| Garcia-Agudo R | 117 | 155 | ≥ 10 | 7 | 40 mcg of recombinant vaccine |
| Kittrakulrat J | 88 | 108 | ≥ 10 | 7 | 40 mcg of recombinant vaccine |
| Hashemi B | 104 | 134 | ≥ 10 | 7 | 40 mcg of recombinant vaccine |
| McNulty C | 37 | 55 | ≥ 10 | 7 | 40 mcg of recombinant vaccine |
| Siddiqui S | 22 | 23 | ≥ 10 | 6 | 20 mcg of recombinant vaccine |
| Seaworth B | 8 | 9 | ≥ 10 | 7 | 20 mcg of recombinant vaccine |
| McNulty C | 29 | 51 | ≥ 10 | 7 | 20 mcg of recombinant vaccine |
| **Recombinant Vaccine** | |  | **i2** | **p-value** |  |
| 10 studies | 75.3% (68.4-81.2) | 0 | 55.02 | 0.018 |  |

**Supplementary Table S4: Pooled Efficacy of Hepatitis B Vaccination in Non-Dialysis CKD Patients: Subgrouped by Study Type**

| **Author** | **N seroconvert** | **N** | **Seroconvert threshold** | **Timing (mo)** | **Vaccine** |
| --- | --- | --- | --- | --- | --- |
| Garcia-Agudo R | 117 | 155 | ≥ 10 | 7 | 40 mcg of recombinant vaccine |
| Hernan-Garcia C | 157 | 173 | ≥ 10 | 7 | 20 mcg of antigen with adjuvanted |
| Fernandez Sanchez-Escalonilla S | 58 | 134 | ≥ 10 | 7 | 20 mcg of antigen with adjuvanted |
| Hashemi B | 104 | 134 | ≥ 10 | 7 | 40 mcg of recombinant vaccine |
| Fabrizi F | 97 | 102 | ≥ 10 | 7 | 20 mcg of antigen with adjuvanted |
| **Cohort** | |  | **i2** | **p-value** |  |
| 5 studies arm | 80.6% (60.8-91.8) | 0.005 | 95.88 | < 0.01 |  |
|  | | | | | |
| **Author** | **N seroconvert** | **N** | **Seroconvert threshold** | **Timing (mo)** | **Vaccine** |
| Siddiqui S | 22 | 23 | ≥ 10 | 6 | 20 mcg of recombinant vaccine |
| Siddiqui S | 9 | 9 | ≥ 10 | 6 | 40 mcg of recombinant vaccine |
| Seaworth B | 11 | 16 | ≥ 10 | 7 | 40 mcg of recombinant vaccine |
| Seaworth B | 8 | 9 | ≥ 10 | 7 | 20 mcg of recombinant vaccine |
| Seaworth B | 12 | 16 | ≥ 10 | 7 | 40 mcg of plasma-derived vaccine |
| Singh NP | 13 | 16 | ≥ 10 | 7 | 40 mcg of recombinant vaccine |
| Singh NP | 16 | 17 | ≥ 10 | 7 | 40 mcg of recombinant vaccine + GCSF |
| Kittrakulrat J | 88 | 108 | ≥ 10 | 7 | 40 mcg of recombinant vaccine |
| McNulty C | 37 | 55 | ≥ 10 | 7 | 40 mcg of recombinant vaccine |
| McNulty C | 29 | 51 | ≥ 10 | 7 | 20 mcg of recombinant vaccine |
| **RCT** | |  | **i2** | **p-value** |  |
| 10 studies arm | 78.2% (69.6-84.9) | < 0.001 | 58.92 | < 0.01 |  |
|  | | | | | |
| **Test for heterogeneity between groups** | | | **Q = 0.11 (p=0.74)** | | |

**Supplementary Table S5: Sensitivity Analysis of Pooled Efficacy of Hepatitis B Vaccination in Non-Dialysis CKD Patients: Only RCT (Exclude Poor Quality Studies)**

| **Author** | **N seroconvert** | **N** | **Seroconvert threshold** | **Timing (mo)** | **Vaccine** |
| --- | --- | --- | --- | --- | --- |
| Garcia-Agudo R | 117 | 155 | ≥ 10 | 7 | 40 mcg of recombinant vaccine |
| Fernandez Sanchez-Escalonilla S | 58 | 134 | ≥ 10 | 7 | 20 mcg of antigen with adjuvanted |
| Hashemi B | 104 | 134 | ≥ 10 | 7 | 40 mcg of recombinant vaccine |
| Fabrizi F | 97 | 102 | ≥ 10 | 7 | 20 mcg of antigen with adjuvanted |
| **Cohort** | |  | **i2** | **p-value** |  |
| 4 studies arm | 76.9% (54.2-90.3) | 0.023 | 95.54 | < 0.001 |  |
|  | | | | | |
| **Author** | **N seroconvert** | **N** | **Seroconvert threshold** | **Timing (mo)** | **Vaccine** |
| Seaworth B | 11 | 16 | ≥ 10 | 7 | 40 mcg of recombinant vaccine |
| Seaworth B | 8 | 9 | ≥ 10 | 7 | 20 mcg of recombinant vaccine |
| Seaworth B | 12 | 16 | ≥ 10 | 7 | 40 mcg of plasma-derived vaccine |
| Kittrakulrat J | 88 | 108 | ≥ 10 | 7 | 40 mcg of recombinant vaccine |
| **RCT** | |  | **i2** | **p-value** |  |
| 4 studies arm | 79.5% (72.1-85.3) | < 0.001 | 0 | 0.567 |  |
|  | | | | | |
| **Test for heterogeneity between groups** | | | **Q = 0.073 (p=0.787)** | | |

**Suppplementary Table S6: COVID-19 Vaccine Effectiveness in Non-Dialysis CKD Patients**

| **Author** | **Vaccine** | **Dose** | **N CKD** | **Antispike IgG+ (BAU/mL)** | **IQR1** | **IQR3** | **Timing** |
| --- | --- | --- | --- | --- | --- | --- | --- |
| Trakarnvanich | ChAdOx1 or inactivated | 2 doses | 12 | 150.13 | 97.12 | 290.65 | 12 weeks |
| Brunwinkler | mRNA vaccine | 2 doses | 160 | 230.3 | 48.3 | 497.6 | 91 days (median time) |
|  | **Pooled mean Antispike IgG+ (BAU/mL)** | | | | **i2** | **p-value** |  |
| 2 paper | 228.386 (152.744, 304.028) | | | | 54.02 | 0.14 |  |
|  |  |  |  |  |  |  |  |
| **Author** | **Vaccine** | **Dose** | **N CKD** | **HR COVID Infection** | **95% CI upper** | **95% CI lower** |  |
| Atiquzzaman | not known type of vaccine | Not vaccinated | 2,418 | Reference |  |  |  |
|  |  | 1 dose | 797 | 0.41 | 0.28 | 0.6 |  |
|  |  | 2 doses | 6,129 | 0.29 | 0.21 | 0.4 |  |
|  |  | 3 doses | 9,506 | 0.22 | 0.13 | 0.38 |  |

**Supplementary Table S7: Pooled Efficacy of Influenza A H1N1 Vaccination in Non-Dialysis CKD Patients: Seroconversion Rates with Subgroup by CKD Stage and Timepoint**

| **Timing** | **Subgroup** | **Pooled percent seroconvert** | **I^2^** | **p-value** | **egger's test** |
| --- | --- | --- | --- | --- | --- |
| **Baseline** |  | 73.067 (65.532, 81.469) | 0 | 0.564 | <0.001 |
|  | CKD G1-3 | 77.501 (64.585, 93.000) | 22.654 | 0.256 |  |
|  | CKD G4-5 | 66.816 (56.784, 78.621) | 0 | 0.918 |  |
|  | 1 dose | 77.279 (67.717, 88.191) | 0 | 0.434 |  |
|  | 2 dose | 64.895 (53.552, 78.642) | 0 | 0.963 |  |
| **4 weeks** |  | 92.758 (87.670, 98.141) | 0 | 0.763 | 0.023 |
|  | CKD G1-3 | 92.053 (84.037, 100.834) | 2.898 | 0.31 |  |
|  | CKD G4-5 | 93.209 (86.686, 100.222) | 0 | 0.678 |  |
|  | 1 dose | 90.094 (82.619, 98.245) | 0 | 0.449 |  |
|  | 2 dose | 94.771 (87.981, 102.085) | 0 | 0.889 |  |
| **8 weeks** |  | 92.758 (87.670, 98.141) | 0 | 0.763 | 0.023 |
|  | CKD G1-3 | 92.053 (84.037, 100.834) | 2.898 | 0.31 |  |
|  | CKD G4-5 | 93.209 (86.686, 100.222) | 0 | 0.678 |  |
|  | 1 dose | 90.094 (82.619, 98.245) | 0 | 0.449 |  |
|  | 2 dose | 94.771 (87.981, 102.085) | 0 | 0.889 |  |
| **20 weeks** |  | 87.124 (81.030, 93.677) | 0 | 0.847 | 0.08 |
|  | CKD G1-3 | 86.052 (74.880, 98.891) | 29.781 | 0.233 |  |
|  | CKD G4-5 | 87.635 (79.851, 96.179) | 0 | 0.907 |  |
|  | 1 dose | 83.138 (74.461, 92.825) | 0 | 0.76 |  |
|  | 2 dose | 90.296 (82.006, 99.425) | 0 | 0.885 |  |

**Supplementary Table S8: Pooled Efficacy of Influenza B Vaccination in Non-Dialysis CKD Patients: Seroconversion Rates with Subgroup by CKD Stage and Timepoint**

| **Timing** | **Subgroup** | **Pooled percent seroconvert** | **I^2^** | **p-value** | **egger's test** |
| --- | --- | --- | --- | --- | --- |
| **Baseline** |  | 41.512 (34.626, 49.767) | 0 | 0.594 | 0.08 |
|  | CKD G1-3 | 48.683 (37.608, 63.019) | 0 | 0.777 |  |
|  | CKD G4-5 | 35.535 (27.539, 45.854) | 0 | 0.869 |  |
|  | 1 dose | 41.834 (30.765, 56.885) | 0 | 0.778 |  |
|  | 2 dose | 40.177 (30.015, 53.779) | 37.22 | 0.203 |  |
| **4 weeks** |  | 80.028 (82.080, 94.406) | 0 | 0.864 | 0.67 |
|  | CKD G1-3 | 89.160 (80.913, 98.248) | 0 | 0.614 |  |
|  | CKD G4-5 | 86.819 (78.486, 96.038) | 0 | 0.683 |  |
|  | 1 dose | 84.974 (77.182, 93.553) | 0 | 0.673 |  |
|  | 2 dose | 91.588 (82.712, 101.416) | 0 | 0.999 |  |
| **8 weeks** |  | 88.959 (81.440, 97.172) | 25.8 | 0.25 | 0.46 |
|  | CKD G1-3 | 92.300 (83.674, 101.815) | 0 | 1 |  |
|  | CKD G4-5 | 86.745 (76.374, 98.526) | 36.39 | 0.194 |  |
|  | 1 dose | 84.305 (73.119, 97.203) | 52.1 | 0.124 |  |
|  | 2 dose | 95.749 (83.589, 109.677) | 0 | 0.994 |  |
| **20 weeks** |  | 86.500 (79.250, 94.413) | 44.7 | 0.108 | 0.12 |
|  | CKD G1-3 | 82.417 (73.960, 91.840) | 0 | 0.908 |  |
|  | CKD G4-5 | 87.844 (77.598, 99.443) | 56.98 | 0.073 |  |
|  | 1 dose | 79.345 (71.864, 87.604) | 0 | 0.671 |  |
|  | 2 dose | 93.888 (86.762, 101.600) | 0 | 0.484 |  |

**Supplementary Table S9: Pooled Efficacy of Herpes Zoster Vaccination in Non-Dialysis CKD Patients**

| **Author** | **Adj. Hazard Ratio** | **95% CI** | **Remark** |
| --- | --- | --- | --- |
| Izurieta HS | 1.03 | 1.00, 1.07 | * |
| Langan SM | 0.51 | 0.35, 0.74 | ** |
|  | **Pooled adjusted hazard ratio** | **p-value** | **I^2^** |
| 2 papers | 0.744 (0.374, 1.478) | 0.398 | 92.556 |

**Adjusted for Demographic factors, socioeconomic conditions, healthcare utilization characteristics, frailty characteristics, functional immunocompromising chronic conditions, and time-varying immunocompromising drugs*

***Adjusted for age, gender, race, low income, immunosuppression, other comorbidities including immune-mediated disorders (systemic lupus erythematosus, inﬂammatory bowel disease and rheumatoid arthritis) and chronic obstructive pulmonary disease, with age and immunosuppression as time-varying covariates*

**Suppplementary Table S10: Pneumococcal Vaccine Seroprotection in Non-Dialysis CKD Patients**

| **Author** | **Vaccine** | **N CKD** | **Time** | **N Seroprotection** | **Notes** |
| --- | --- | --- | --- | --- | --- |
| Wall | Pneumovax PPV23 | 23 | Baseline | 16 |  |
|  |  |  | 28 days | 21 | 8 of 12 serotypes protection and two fold increase of titer |
| Fuchshuber | Pneumovax PPV23 | 11 | Baseline | 1 |  |
|  |  |  | 4 weeks | 10 | titer > 200 or two-fold increase |
|  |  |  | 6 months | 8 |  |
|  |  |  | 1 year | 6 |  |

**Supplementary Figure S1: Funnel Plot of Studies Reporting HBV Vaccine Efficacy in CKD-ND**

**
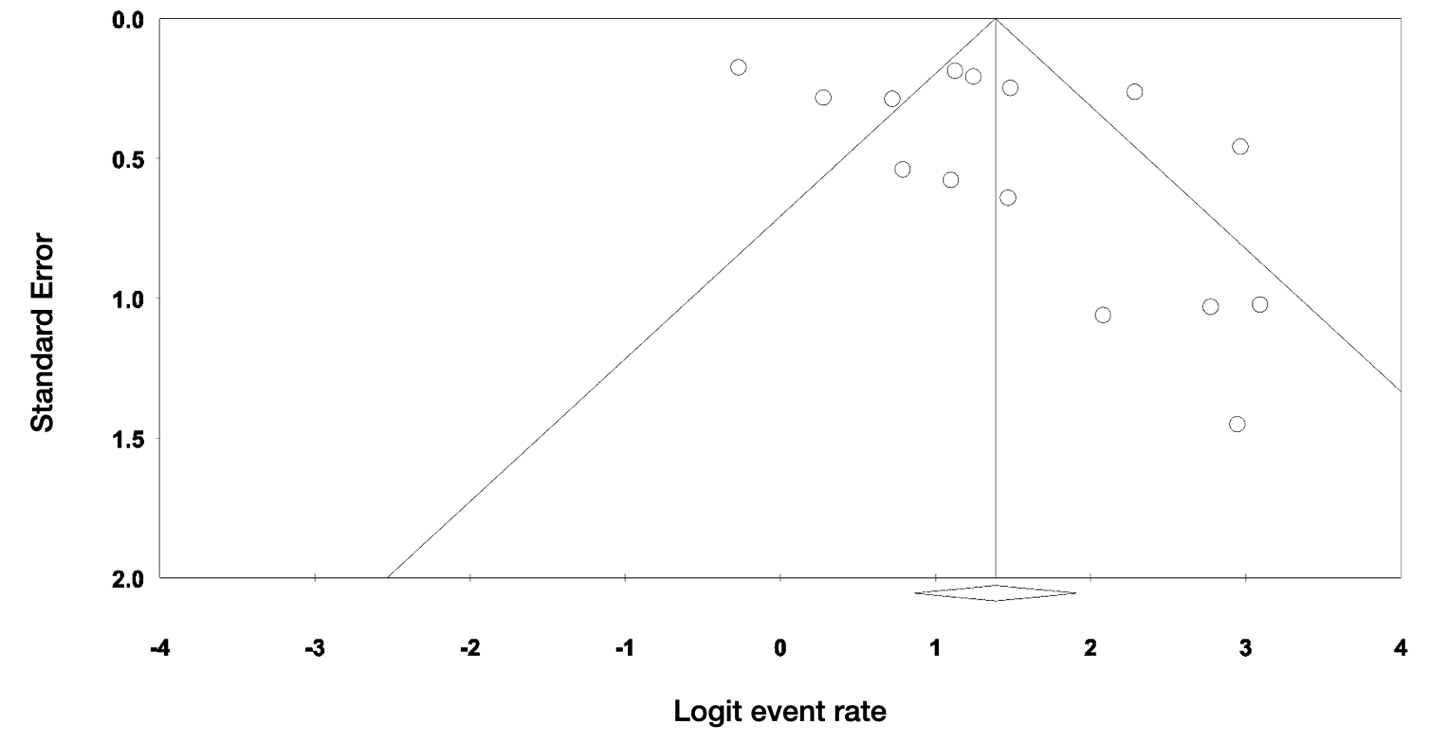
**
